# A phase Ib study of interleukin-2 plus pembrolizumab for patients with advanced melanoma

**DOI:** 10.3389/fonc.2023.1108341

**Published:** 2023-02-09

**Authors:** Ann W. Silk, Brendan Curti, Jennifer Bryan, Tracie Saunders, Weichung Shih, Michael P. Kane, Phoebe Hannon, Christopher Fountain, Jessica Felcher, Andrew Zloza, Howard L. Kaufman, Janice M. Mehnert, David F. McDermott

**Affiliations:** ^1^ Dana-Farber Cancer Institute, Boston, MA, United States; ^2^ Harvard Medical School, Department of Medicine, Boston, MA, United States; ^3^ Rutgers Cancer Institute of New Jersey, New Brunswick, NJ, United States; ^4^ Robert Wood Johnson Medical School, New Brunswick, NJ, United States; ^5^ Earle A. Chiles Research Institute, Providence Cancer Institute, Portland, OR, United States; ^6^ Virginia Mason Cancer Institute, Seattle, WA, United States; ^7^ Beth Israel Deaconess Medical Center, Boston, MA, United States; ^8^ Rush University Medical Center, Department of Internal Medicine, Chicago, IL, United States; ^9^ Ankyra Therapeutics, Boston, MA, United States; ^10^ Massachusetts General Hospital, Boston, MA, United States; ^11^ Laura and Isaac Perlmutter Cancer Center, New York University Langone Medical Center, New York, NY, United States

**Keywords:** melanoma, interleukin-2, cytokine, pembrolizumab, combination immunotherapy

## Abstract

**Introduction:**

High-dose interleukin-2 (HD IL-2) and pembrolizumab are each approved as single agents by the U.S. F.D.A. for the treatment of metastatic melanoma. There is limited data using the agents concurrently. The objectives of this study were to characterize the safety profile of IL-2 in combination with pembrolizumab in patients with unresectable or metastatic melanoma.

**Methods:**

In this Phase Ib study, patients received pembrolizumab (200 mg IV every 3 weeks) and escalating doses of IL-2 (6,000 or 60,000 or 600,000 IU/kg IV bolus every 8 hours up to 14 doses per cycle) in cohorts of 3 patients. Prior treatment with a PD-1 blocking antibody was allowed. The primary endpoint was the maximum tolerated dose (MTD) of IL-2 when co-administered with pembrolizumab.

**Results:**

Ten participants were enrolled, and 9 participants were evaluable for safety and efficacy. The majority of the evaluable participants (8/9) had been treated with PD-1 blocking antibody prior to enrollment. Patients received a median of 42, 22, and 9 doses of IL-2 in the low, intermediate, and high dose cohorts, respectively. Adverse events were more frequent with increasing doses of IL-2. No dose limiting toxicities were observed. The MTD of IL-2 was not reached. One partial response occurred in 9 patients (11%). The responding patient, who had received treatment with an anti-PD-1 prior to study entry, was treated in the HD IL-2 cohort.

**Discussion:**

Although the sample size was small, HD IL-2 therapy in combination with pembrolizumab appears feasible and tolerable.

**Clinical trial registration:**

ClinicalTrials.gov, identifier NCT02748564.

## Introduction

Since the 1990s, it has been known that responses to high dose interluekin-2 (HD IL-2) in melanoma patients are low in frequency but exceptionally durable (1, 2). According to an analysis of 270 melanoma patients who participated in 8 clinical trials between 1985 and 1993, the overall response rate was 16%; importantly, nearly half (47%) of the responding patients survived 5 years or more (1). Due to the associated toxicity, mainly hypotension and capillary leak syndrome, HD IL-2 therapy is administered in the inpatient setting. Most patients are admitted for 5-6 days in the hospital to receive up to 14 doses of HD IL-2 per cycle, as tolerated. In contrast to HD IL-2, immune checkpoint inhibitors, especially PD-1 inhibitors, are generally well-tolerated and have favorable toxicity profiles. Therefore, in the modern era of front-line immune checkpoint inhibitor therapies, the application of HD IL-2 has become more limited, and it is typically reserved as a salvage therapy for patients whose cancer fails to respond to PD-1 inhibition.

Salvage therapy with HD IL-2 has been studied in patients with immune checkpoint-refractory disease in a registry called PROCLAIM, in which data were captured prospectively during HD IL-2 treatment. Among melanoma patients in the PROCLAIM registry who were treated with HD IL-2 after prior treatment with ipilimumab or an anti-PD1 antibody, the response rates were 21% (11 of 52 patients) and 22.5% (9 of 40 patients), respectively (3, 4). These response rates are comparable to historical controls (1, 2); these data suggest that HD IL-2 treatment retains its effectiveness in patients whose melanoma is refractory to ipilimumab or anti-PD-1 treatment. According to the PROCLAIM registry data, toxicity was manageable, although 1 of 57 patients who received prior anti-PD-1 developed pneumonitis requiring steroid therapy, suggesting that HD IL-2 can re-activate checkpoint inhibitor-like toxicity on occasion (4). Patients received an average of 8-9 doses of IL-2 per cycle, as expected, indicating that overall, these patients tolerated HD IL-2 as well as historical controls.

Concurrent therapy with immune checkpoint inhibitor plus HD IL-2 has been explored in clinical trials for treatment of melanoma and renal cell carcinoma recently, based on reported synergy between IL-2 based therapy and checkpoint blockade in preclinical models (5). We previously conducted a small prospective clinical trial of concurrent HD IL-2 plus ipilimumab (6). We found that 1 of 9 patients with melanoma responded to this combination. There were no new safety signals observed; however, expected IL-2-related side effects such as liver and kidney injury were more prolonged than usual, and systemic corticosteroids were required to treat immune-related adverse events in one-third of the subjects. Combining IL-2 with PD-1 is more attractive than ipilimumab, owing to the better safety profile and antitumor activity of PD-1 blockade in patients with melanoma. It is known that IL-2 production can be suppressed by the actions of the PD-1 checkpoint (7), and that modulation by PD-1 inhibitors can reverse this anergy (5). In humans with melanoma undergoing anti-PD-1 therapy, response is correlated with proliferation of intra-tumoral lymphocytes (8). We hypothesized that these tumor-infiltrating lymphocytes could be stimulated by IL-2 therapy. In the present study, we treated patients with unresectable or metastatic melanoma with the standard dose of pembrolizumab and escalating doses of IL-2 to determine the safety of the combination and to select a dose of IL-2 for further study in combination with anti-PD-1 therapy.

## Methods

### Patient selection

Adults with histologically confirmed unresectable stage III and IV melanoma and ECOG performance status 0-1 and normal cardiopulmonary and renal function were enrolled at three academic centers between 2017 and 2019. Main exclusion criteria were ocular melanoma, active brain metastases, active autoimmune disease, and use of concurrent systemic immunosuppressive therapy. Patients who had received prior treatment with IL-2 were excluded. All other prior therapies, including pembrolizumab, were allowed. The clinical protocol was approved by all local institutional review board (IRB) prior to patient accrual. All patients gave written informed consent to be treated.

### Design

In this Phase Ib study, cohorts of 3 patients were treated with pembrolizumab (200 mg IV every 3 weeks) and escalating doses of IL-2 (6,000 or 60,000 or 600,000 IU/kg IV bolus every 8 hours). The primary endpoint was the (MTD) of IL-2 when co-administered with pembrolizumab. Ten participants were enrolled, and 9 participants were evaluable for safety and efficacy.

### Treatment

**Figure 1:**
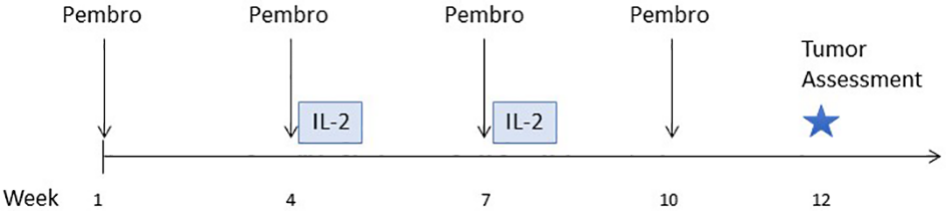
Study schema. Treatment began with pembrolizumab only for the first cycle. IL-2 therapy (2 cycles of up to 14 doses each) was given immediately following the second and third cycles of pembrolizumab.

Patients received pembrolizumab (200 mg flat dose IV) every 3 weeks, and a course IL-2 beginning with the second cycle of pembrolizumab (**Figure 1**). Each course of IL-2 consisted of 2 cycles of up to 14 doses each, as tolerated. After up to 3 courses of IL-2 plus pembrolizumab, patients could continue to receive pembrolizumab monotherapy for up to two years. Cohorts of 3 patients received IL-2 in a low, medium, or high dose (6,000, 60,000, or 600,000 IU/kg) IV bolus every 8 hours for up to 14 doses, as tolerated. A physical examination and laboratory tests (including CBC with differential and comprehensive metabolic profile and thyroid tests) were done at screening and every 3 weeks. Safety assessments were performed daily during hospitalization for IL-2 therapy. Adverse events (AEs) were evaluated and graded using NCI Common Toxicity Criteria v4.0. Dose reductions were not permitted for either drug. Both drugs were held and/or discontinued for high grade autoimmune toxicity. No intra-patient dose escalation was allowed. Imaging for tumor assessment was performed every 12 weeks. Response was assessed using RECIST criteria version 1.1 (9). Patients were considered evaluable for safety and efficacy if they received at least one dose of each study drug. The primary endpoint was defined as the (MTD) of IL-2 in combination with pembrolizumab. More than half of patients treated with high-dose IL-2 experience transient grade 3 toxicities; thus, dose limiting toxicity (DLT) was defined as any treatment-related grade 3 event that occurred during the first 6 weeks of study treatment and did not resolve to grade 2 or less within 14 days of onset. In addition, any treatment-related grade 4 or 5 toxicity that occurred during the first 6 weeks of study treatment was considered a DLT, with exceptions for specific reversible grade 4 events that are expected with IL-2 therapy: hypotension, decreased urine output, pulmonary edema, cytokine release syndrome and venous access complications. Descriptive statistics were used for clinical outcome and safety reporting. Blood samples were collected on all subjects for correlative analysis and tumor biopsies were optional. The protocol included a plan for an expansion to treat 3 additional subjects at the MTD for a total of 6 subjects treated at the MTD, and a Phase 2 portion with a target sample size of 48 patients; however, the sponsor terminated the study due to slow enrollment before the Phase 2 portion could begin.

## Results

### Patient characteristics

**Figure 2:**
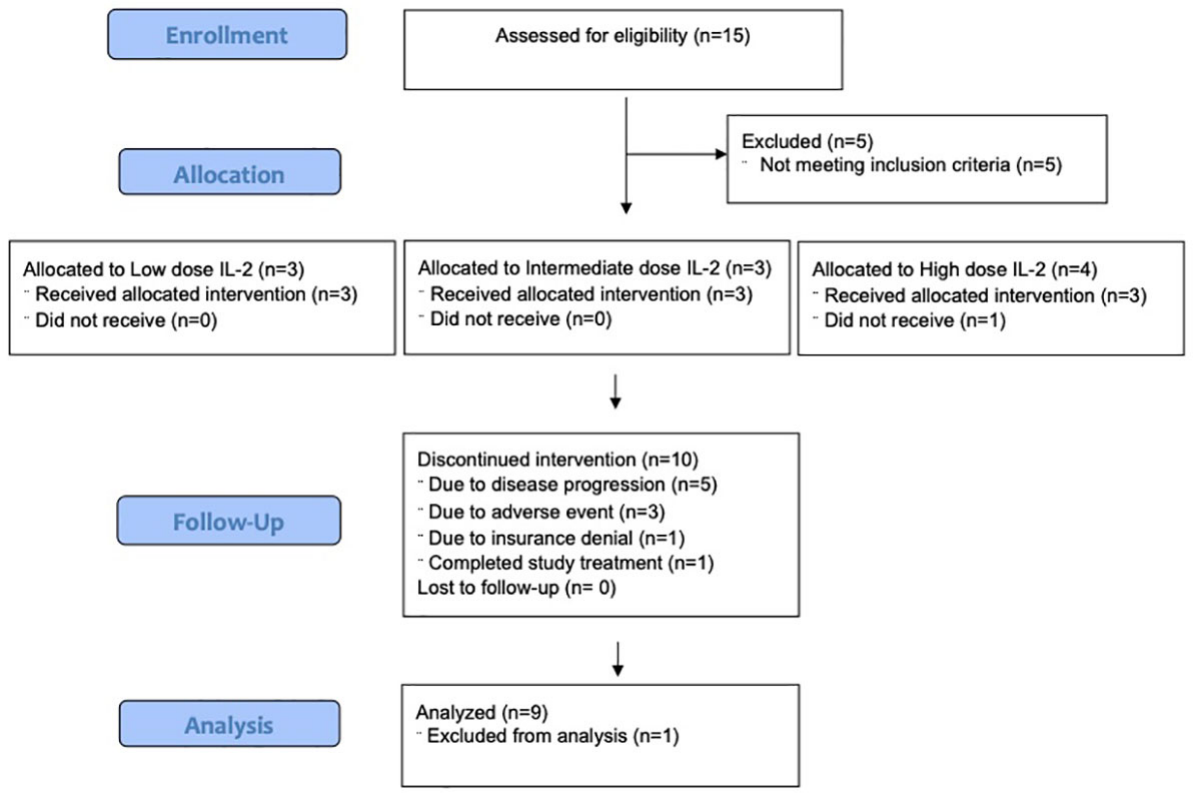
CONSORT diagram.

Fifteen patients were consented. Five patients did not meet eligibility criteria. Ten patients were enrolled, and 9 patients were evaluable for safety and efficacy (**Figure 2**). One patient began the first cycle of treatment but was unable to receive IL-2 due to denial of health insurance coverage, so the subject was removed from study. Baseline patient characteristics of the 10 patients who enrolled are summarized in **Table 1**. Most patients (9/10) received anti-PD-1 prior to study entry. Patients received a median of 42, 22, and 9 doses of IL-2, and a median of 8, 5, and 2 doses of pembrolizumab in the low, intermediate, and high dose cohorts, respectively (**Table 2**). One patient completed 2 years of treatment on study.

**Table 1:** Baseline characteristics of 10 patients who enrolled on the study.

Gender	F	4 (40%)
	M	6 (60%)
Race	White	10 (100%)
Ethnicity	Non-Hispanic	10 (100%)
Age	30-39	3 (30%)
	40-49	3 (30%)
	50-59	1 (10%)
	60-69	2 (20%)
	70-79	1 (10%)
Liver metastases	Present	3 (30%)
	Absent	7 (70%)
Elevated LDH	≥ 250 UI/mL	3 (30%)
	< 250 UI/mL	7 (70%)
Prior treatment	Pembrolizumab or nivolumab	9 (90%)
	Ipilimumab	6 (60%)
	Interferon-alpha	1 (10%)
	Targeted therapy	1 (10%)
	Radiation therapy	5 (50%)

**Table 2:** Treatment administration.

IL-2 Dose Level	Number of doses of pembrolizumab administered (median, range)	Number of doses of IL-2 administered (median, range)
6,000 IU/kg	8 (2–13)	42 (14–52)
60,000 IU/kg	5 (3–86)	22 (15–24)
600,000 IU/kg	2 (1–8)	9 (8–18)

### Safety

There were no treatment-related deaths. Six of the 9 evaluable participants (67%) experienced 19 grade 3 or 4 treatment-related adverse events (**Table 3**). There were more adverse events in the higher doses of IL-2 (**Table 3**). There were no (DLTs). Three patients discontinued both study drugs due to adverse events, which included a patient with nausea at the intermediate dose of IL-2 (60,000 IU/kg), and a patient with rash, which occurred at the high dose of IL-2 (600,000 IU/kg). A third patient, also treated at the high dose of IL-2, discontinued due to a combination of adrenal insufficiency and dyspnea not otherwise specified, and was treated with reater than 40 mg prednisone daily and inhaled steroids. No patient required steroids greater than 40 mg daily to treat an adverse event.

**Table 3:** Grade 3 and 4 adverse events related to one or both study drugs, by IL-2 dose level.

IL-2 Dose Level	Number of patients who experienced treatment-related Grade 3 or 4 AE	Type of AEs
6,000 IU/kg	0/3 (0%)	•None
60,000 IU/kg	2/3 (67%)	•Acute kidney injury •Lymphocyte count decreased
600,000 IU/kg	3/3 (100%)	•Diarrhea •Hypotension •Thromboembolic event •Maculopapular rash •Lymphocyte count decreased •Hyponatremia •Hypophosphatemia
**Total**	**6/9 (67%)**	

### Efficacy

**Figure 3:**
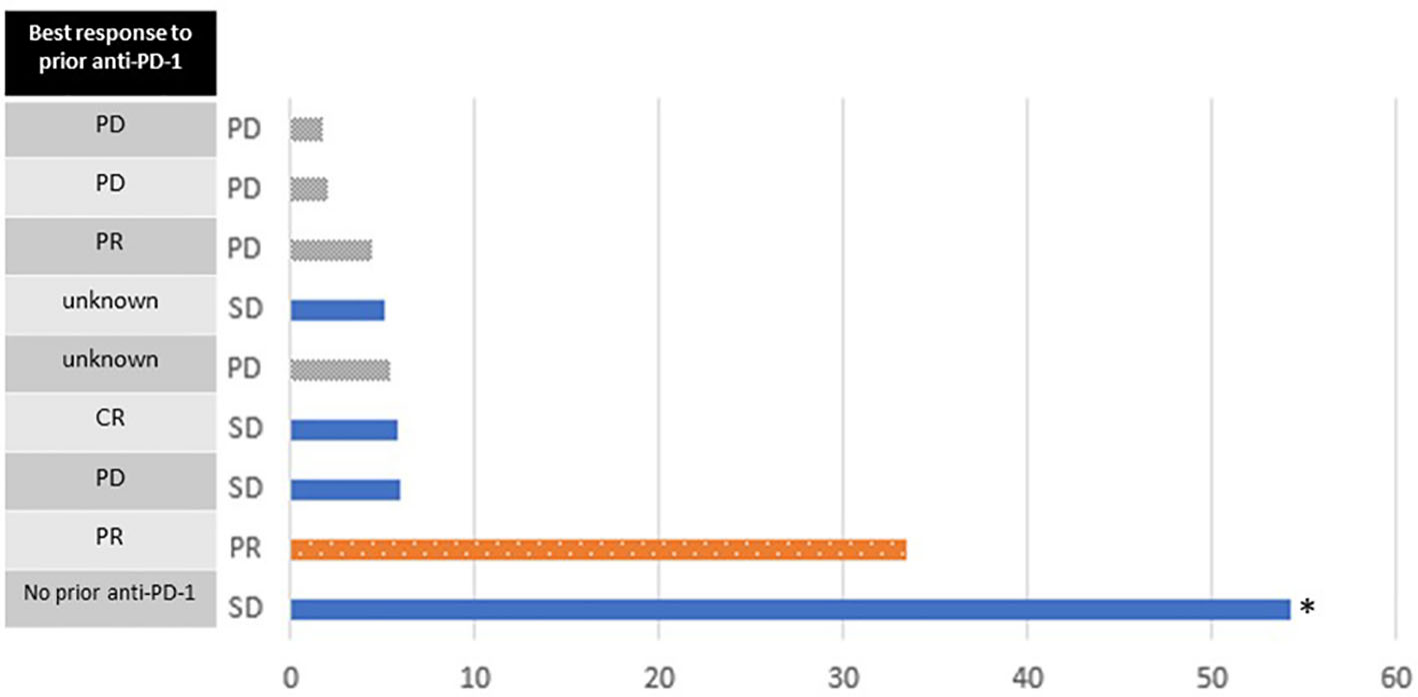
Swimmer plot of progression-free survival (months). Bars are colored according to the best response on study. Best response to prior anti-PD-1 treatment is shown on the left. The asterisk indicates data censored at the time of last follow-up.

In the 9 evaluable participants, there was 1 partial response (11%; **Table 4**). Four participants had stable disease (44%) and 4 had progressive disease (44%). The partial response was observed in the patient who discontinued high dose IL-2 due to grade 3 rash, as above. His previous treatments consisted of nivolumab followed by anti-LAG-3 therapy, which ended 10 months and 1 month prior to study entry, respectively. The median follow-up time was 20.4 months. Two patients had durable progression-free survival, lasting 33 and 54+ months (**Figure 3**). At the time of this analysis, 8/9 participants have expired. The median overall survival (OS) was 20.4 months, with 1-year and 2-year OS rates of 6/9 (67%) and 4/9 (44%), respectively. One participant who had a reduction in tumor burden that was classified as stable disease remains alive 4.5 years after study entry. He had received ipilimumab in the adjuvant setting, but he did not receive treatment with PD-1 checkpoint antibodies prior to study entry. Only one fresh tumor biopsy was collected. Although blood samples were collected on all patients, samples from only 5 subjects could be located at the time of data analysis. Thus, no correlative samples were analyzed.

**Table 4:** Best overall response rate by IL-2 dosing cohort.

IL-2 Dose Level	Response Rate
6,000 IU/kg	0/3 (0%)
60,000 IU/kg	0/3 (0%)
600,000 IU/kg	1/3 (33%)
**Total**	**1/9 (11%)**

## Discussion

In this dose-escalation study of 9 evaluable patients, we demonstrated that treatment with a range of doses of IL-2 concurrent with pembrolizumab is feasible. No new safety signals were identified. The number of patients who experienced one or more serious treatment-related AEs increased with increasing doses of IL-2, which was an expected finding. There was one response observed (11%) in a patient with PD-1 refractory melanoma who was treated with HD IL-2. Another patient had stable disease that was durable for more than 4.5 years (censored at the time of last follow-up), without the need for further treatment. However, as he did not have prior PD-1 therapy, the control of his disease may be attributable to the pembrolizumab alone. This exploratory study was designed to determine the safety and feasibility of combination IL-2 and pembrolizumab, but the sample size was too small to make definitive conclusions on clinical responses. Patients in this study generally skewed younger than the average melanoma patient, and all but one patient had received anti-PD-1 therapy prior to study entry, reflecting the typical patient population that would be considered for salvage HD IL-2 therapy in the modern era.

There are two key problematic features of native IL-2 as a therapeutic agent, which are the short half-life, and the potent activation of regulatory T cells, especially at lower doses. Low doses of the native form of IL-2 have been reported to preferentially expand regulatory T cells (Tregs) which is mediated by the alpha subunit (CD25) of the IL-2 receptor. In a B16-F10 melanoma mouse model, treatment with high and intermediate doses of IL-2 inhibited tumor outgrowth compared to placebo, but treatment with low−dose IL-2 allowed more tumor outgrowth than the placebo (10). An expansion of Tregs was observed in the tumor with low dose IL-2, but not with intermediate or high dose IL-2, possibly owing to the fact that there is abundant expression of high-affinity IL-2 trimeric (CD25–CD122–CD132) receptors on Tregs as compared to effector T cells (11). In the current study, we observed that patients treated at lower doses were able to tolerate more doses of IL-2 (**Table 2**); however, no responses were observed at the low or intermediate doses of IL-2.

New variants of IL-2 designed to preferentially target CD8+ effector cells are in clinical testing (12, 13). Pegylation is another popular strategy to both extend the half-life of IL-2 and to avoid CD25 signaling. The pegylation confers selectivity for CD122 and CD132, also called the beta and gamma subunits (14, 15). Despite promising preclinical work, the addition of bempegaldesleukin to nivolumab did not result in improvement in response rate or progression-free survival compared to nivolumab alone in a randomized Phase 3 trial in melanoma patients (16, 17). Recent work in a chronic viral infection model demonstrated that IL-2 influences the differentiation of stem-like CD8^+^ T cells, creating distinct sets of PD-1^+^CD8^+^ effector T cells with superior antiviral activity as compared to PD-1 blockade treatment alone. CD25 engagement by IL-2 was shown to be critical for synergy between IL-2 and anti-PD-1 that was observed in these experiments (18), suggesting that the contribution of the alpha subunit of the IL-2 receptor is dynamic and complex, which may provide insight into the difficulty with targeting the IL-2 receptor therapeutically thus far.

## Data availability statement

The corresponding author has left the institution; dataset can be available upon request to co-author TS, Director of Clinical Trials Office. Requests to access the datasets should be directed TS, tks13@cinj.rutgers.edu.

## Ethics statement

The studies involving human participants were reviewed and approved by Institutional Review Board at Rutgers University. The patients/participants provided their written informed consent to participate in this study.

## Author contributions

AS, BC, HK, JM, WS, and DM, contributed to the study concept, design, analysis and interpretation of data. AS and DM drafted the manuscript. AS, TS, WS, and DM analyzed and interpreted data and drafted the manuscript. AS, BC, JB, PH, CF, JF, HK, JM, and DM were involved in data acquisition. All authors contributed to the article and approved the submitted version.
